# Synthesis of solid sodium silicate from waste glass and utilization on one-part alkali-activated materials based on spent oil filtering earth

**DOI:** 10.1007/s11356-024-33368-w

**Published:** 2024-05-02

**Authors:** Pedro Delgado-Plana, Salvador Bueno-Rodríguez, Luis Pérez-Villarejo, Dolores Eliche-Quesada

**Affiliations:** 1 Department of Chemical, Environmental, and Materials Engineering, Higher Polytechnic School of Jaén, University of Jaén, Campus Las Lagunillas S/N, 23071 Jaén, Spain; 2 Centre for Advanced Studies in Earth Sciences, Energy and Environment (CEACTEMA), University of Jaén, Campus Las Lagunillas, S/N, 23071 Jaén, Spain; 3 Department of Chemical, Environmental, and Materials Engineering, Higher Polytechnic School of Linares, University of Jaen, Campus Científico-Tecnológico, Cinturón Sur S/N, Linares, 23700 Jaén, Spain

**Keywords:** Waste-based activator, Thermochemical synthesis, Microstructural study, One-part alkali–activated materials, Mechanical strength, Solid activator

## Abstract

Alkali activated materials (AAMs) commonly known as geopolymers are considered ecofriendly substitutes for Portland cement. However, these materials still have a significant environmental impact, owing mainly to the use of activators based on commercial chemical products. In this sense, this research focuses on the production and use of waste glass–derived activators AAMs as an alternative to commercial activators. Using a thermochemical synthesis method, activator compositions were systematically designed to achieve predefined activator modulus (Ms = SiO_2_/Na_2_O = 0.5; 1.0 and 1.5). These alternative activators were studied by XRD, FTIR and SEM techniques. Additionally, one-part AAMs were manufactured using spent oil filtration earth (SOFE) as precursor and activator with optimum modulus Ms = 1.0. The influence of the Na_2_O dosage was studied (10; 20 and 30 g of Na_2_O per every 100 g of SOFE) as well as the influence of the activator modulus maintaining the optimum dosage of 20 g Na_2_O per 100 g of SOFE. As a control, two-part AAMs were also synthetized with the optimum dosage and modulus employing commercial activators (NaOH + Na_2_SiO_3_ solution). Results indicate that the modulus of the alternative activator and especially the Na_2_O dosage have a significant influence on the technological properties of AAMs based in SOFE, with an optimum compressive strength (35.8 MPa) for the addition of 20 g of Na_2_O per every 100 g of SOFE using activator with modulus Ms = 1.0. This research embodies a sustainable approach to AAM production and suggests waste glass as a valuable raw material for sodium silicate synthesis intended for the one-part activation of spent filtering earth from the agri-food industry, aligning with the principles of circular economy and sustainable development goals.

## Introduction

The construction industry is recognized as a major contributor to global carbon dioxide emissions, in part due to the prevalent use of Portland cement, which accounts for around 7% of total fossil fuel–related carbon dioxide emissions worldwide (Nwankwo et al. 2020). In this sense, as the world is dealing with the challenge of climate change, alternative construction materials and practices have gained significant attention, and alkali-activated materials (AAMs) have emerged as a promising and sustainable alternative to conventional cementitious materials.

AAMs are inorganic, aluminosilicate-based materials that exhibit remarkable properties, making them a compelling substitute for Portland cement. They can be formed through the alkaline activation of aluminosilicate precursors from natural sources as metakaolin (Samuel et al. 2020; Longhi et al. 2022) and clays (Hamdi et al. 2018; Liew et al. 2016) or from wastes such as fly ash (Li et al. 2022; Wang et al. 2023), slags (Shi et al. 2018; Jin et al. 2014), agri-food residues (Hossain et al. 2021) and other wastes.

Filtering earths are mainly formed by diatomaceous earth which is a natural sedimentary material composed of fossilized diatoms (Ha et al. 2015) and considered a low-cost abundant mineral (Bagci et al. 2017). In this context, one of the most widely recognized applications of diatomite is as a filtration material. Specifically, diatomite-based filtering earth has found significant application in the oil industry as an efficient filtration medium (Michen et al. 2011) and has demonstrated its effectiveness in the removal of specific contaminants (Jian et al. 2013; Wang et al. 2019). Nevertheless, the replacement of spent oil filtering earth residue (SOFE) implies significant challenges in terms of waste management. Once used, the spent material becomes a wet and oily waste material that requires specialized handling and disposal methods to ensure compliance with waste regulations and minimize any potential adverse effects on the environment.

As an alternative to disposal, SOFE can be used as raw material in the manufacture of AAMs due to its typically high content in silica and moderate content in alumina. However, it is also essential in this case to address a critical aspect of AAMs. They are undoubtedly greener than traditional cementitious materials, with a reduction in equivalent CO_2_ emissions between 26 and 80% (Elmesalami and Celik 2022; Duxson et al. 2007) but still have a certain global warming contribution, which largely depends on the activators used in their production. The kind and amount of activator employed in the synthesis of the AAMs dramatically influence their properties (Navarro et al. 2022; Bernal 2015) as well as their global warming potential. According to some studies, the contribution of the activator accounts for up to 80% of the total global warming potential contribution of the AAMs when a life cycle assessment is carried out (Garces et al. 2021).

Sodium silicate is, in conjunction with sodium hydroxide, a widely used activator, well known for enhancing the mechanical properties of AAMs. However, it is particularly polluting and its usage should be minimized. This is attributed to the production process, involving the high-temperature calcination of sodium carbonate and quartz in furnaces at approximately 1500 °C, releasing CO_2_ as a byproduct (Bernal et al. 2012). Subsequently, this solid must be dissolved in water under moderately elevated temperature and pressure for its later application as part of an activating solution. As a result, the embodied energy of sodium silicate commercial solution is considered to be around 5 MJ/kg, with carbon dioxide emissions of 1.5 kg CO_2_/kg (Rajan and Kathirvel 2021). For this reason, there has been a growing emphasis on the development and utilization of alternative activators for AAM manufacture (Mendes et al. 2021; Kumar et al. 2023). One notable approach involves the synthesis of sodium silicate using NaOH in combination with different residual sources of silica. In particular, one of the residues more extensively used is waste glass. In Europe, 26% of glass from the container sector is not recycled, amounting to approximately 5.5 Mt per year. This material is typically used as an additive in cement, fibreglass manufacturing and other purposes. One of the reasons why a significant amount of glass is not recyclable is due to the presence of dyes, heavy metals and even residues from the ceramic industry (Samarakoon et al. 2021). Hence, waste glass can be regarded as a valuable byproduct for utilization as a raw material in other industries, and it is consistently generated worldwide. In this sense, waste glass can be used as a source of silica in the synthesis of sodium silicate under several different methods that can be classified in hydrothermal synthesis and thermochemical synthesis methods.

Hydrothermal synthesis has been widely studied and employed to obtain sodium silicate–rich solutions from various residues. Materials often utilized as a source of silicon include rice husk ash (Rajan and Kathirvel 2021), waste glass (Mendes et al. 2022) and diatomite (Font et al. 2018). However, in recent years, new sources like municipal solid waste ashes (Alam et al. 2019) and kaolinite (Rao et al. 2022) have been also utilized. Hydrothermal synthesis methods involve bringing the solid source of silica into close contact with a sodium hydroxide solution (Dadsetan et al. 2022) and, optionally, heating the mixture at relatively low temperatures by external means, typically below 90 °C, during times that often range between 6 and 24 h (Tong et al. 2018). Common variables in this method include using a heated reactor or closed vessels (Font et al. 2018) (sometimes within an oven), the use of a stirrer, controlling the duration and temperature of the treatment and preparation of the residue-NaOH blend. The viscous liquid obtained from this process is expected to contain a certain concentration of sodium silicate. The liquid can be utilized as an activator after removing suspended solids through vacuum filtration or directly used as produced. In contrast, thermochemical synthesis methods are based on the mixing of solid NaOH and the silica-containing residue and the subsequent treatment at moderately high temperatures (150–300 °C) during periods of time usually ranging from 1 to 3 h (Vinai and Soutsos 2019). Other authors used higher temperatures (500, 600 and 700 °C) during 2 h (Marin et al. 2023). Some variations in this method stem from the aggregation state of the residue and the NaOH and the way that they are mixed. Some examples include “mechanical dry mixing” in a ball mill or “wet mixing” that involves the addition of a small amount of water to the mixture to facilitate homogenization. All these methods related to thermochemical synthesis present some advantages such as no further residues are obtained, the modulus Ms and the Na_2_O content of the activator are predictable and the possibility to work via one part. This last aspect is considered strategic since one-part AAMs are safer and easier to handle, so they are gaining attention in both scientific and industrial sectors (Ren et al. 2021).

## Significance of the research

In this study, alternative solid activators were produced under the thermochemical synthesis method and used in the manufacture of one-part AAMs based on spent oil filtering earth (SOFE) alternatively activated. This research addresses the activation of a residual material, SOFE, using alternative waste-derived activators from a comprehensive approach, from optimizing the activator manufacture process to adjusting its dosage for the production of AAMs with enhanced mechanical properties.

Through this approach, this research aims to address three critical aspects:

- Determining the ideal Ms ratio for the thermochemical synthesis of sodium silicate from NaOH and waste glass
- Establishing the optimum dosage of the ideal activator for AAMs with superior technological properties
- Clarifying the advantages of using this ideal alternative solid activator compared with equivalent dosages of alternative activators with different moduli or commercially available activators with equivalent modulus and dosage

The novelty of this study stems from this global approach and the precise design of NaOH-waste glass mixtures based on chemical criteria and calculations, enabling the generation of predefined activator quantities with a specific modulus Ms. In addition, since the entire resulting material serves as a solid activator, the exact dosages of these activators to achieve a certain supply of Na_2_O can be easily calculated under this method. It allows the comparison of the manufacture of AAMs with other processing routes using different alternative or commercial activators under the same dosage conditions.

## Materials and methods

### Experimental design

This research can be divided into three distinct phases, according to the objectives established in the “Significance of the research” section. The initial phase focused on the development of solid alternative activators using waste glass and sodium hydroxide, with variations in the activator modulus (mol SiO_2_/mol Na_2_O), aimed at identifying the optimal value (Ms = 0.5, 1.0, 1.5). The theoretical suitability of the manufactured activators was evaluated utilizing analytical techniques such as X-ray diffraction (XRD) and attenuated total reflectance Fourier-transform infrared (ATR-FTIR) spectroscopy, as elaborated upon in subsequent sections.

In the second stage, three batches of one-part AAMs were synthesized by blending the precursor SOFE, the optimum activator (Ms = 1.0) at various dosages (10, 20, 30 g Na_2_O per 100 g of precursor) and distilled water. This phase was aimed at determining the ideal dosage of the optimum activator to achieve AAMs with the most desirable technological properties.

During the third phase, to validate the suitability of the selected activator and dosage, two more sets of samples were manufactured to be compared with those made with the ideal dosage of the optimum activator:

1. Two-part AAMs manufactured using the same precursor and an activating solution made from distilled water, NaOH pellets (Panreac, 98%) and commercial sodium silicate solution (Panreac. 29.2% SiO_2_; 8.9% Na_2_O and 61.9% H_2_O), maintaining a consistent addition of Na_2_O and Ms ratio.
2. Samples produced with alternative solid activators, where the Ms ratio was not considered optimal (Ms = 0.5, 1.5) but the Na_2_O addition remained constant.

**Fig. 1:**
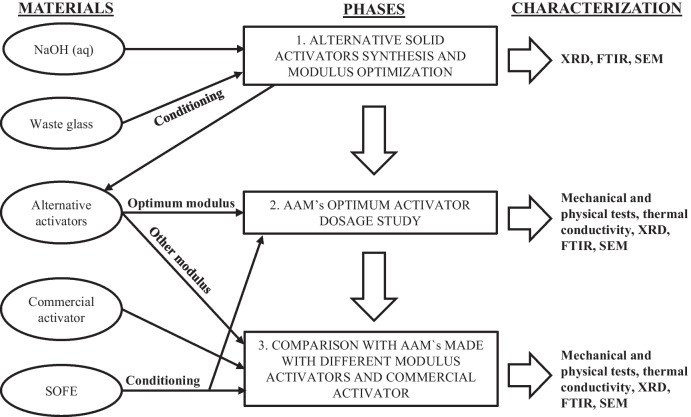
Experimental design

The experimental design is exposed in Fig. 1.

### Raw materials characterization and pretreatment

#### Spent oil filtering earth (SOFE)

SOFE residue was collected from the olive oil processing industry Aceites Coosur in Vilches (Andalusia, Spain). The residue, as received, presented high fat and moisture content so it underwent a pre-processing phase prior to its utilization as a precursor material in AAM production. Initially, the waste material was subjected to an oven treatment at 105 °C until a consistent weight was achieved, effectively eliminating moisture content.

**Fig. 2:**
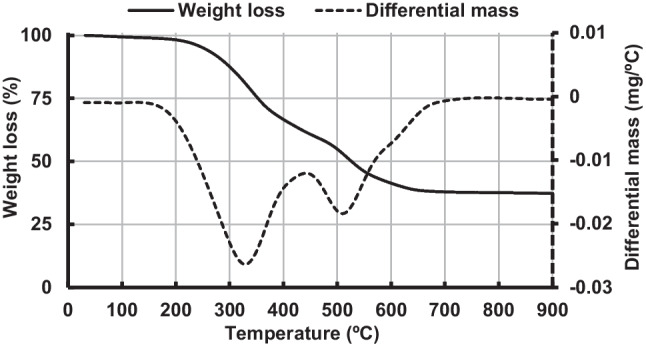
Thermogravimetric analysis (TGA) and derivative thermogravimetry (DTG) of SOFE raw material

Subsequently, a heat treatment at 700 °C for 2 h was conducted, employing a heating ramp of 10 °C per minute, aimed at eliminating the organic matter. The optimal temperature for the removal of the organic fraction through calcination was established by means of a thermogravimetric analysis conducted on a sample using a Mettler Toledo thermal analyzer model TGA/DSC 1. This analysis employed an airflow of 50 mL/min and involved heating at a rate of 10 °C/min within a temperature range spanning from 30 to 900 °C. The results of thermogravimetric analysis are shown in Fig. 2.

**Fig. 3:**
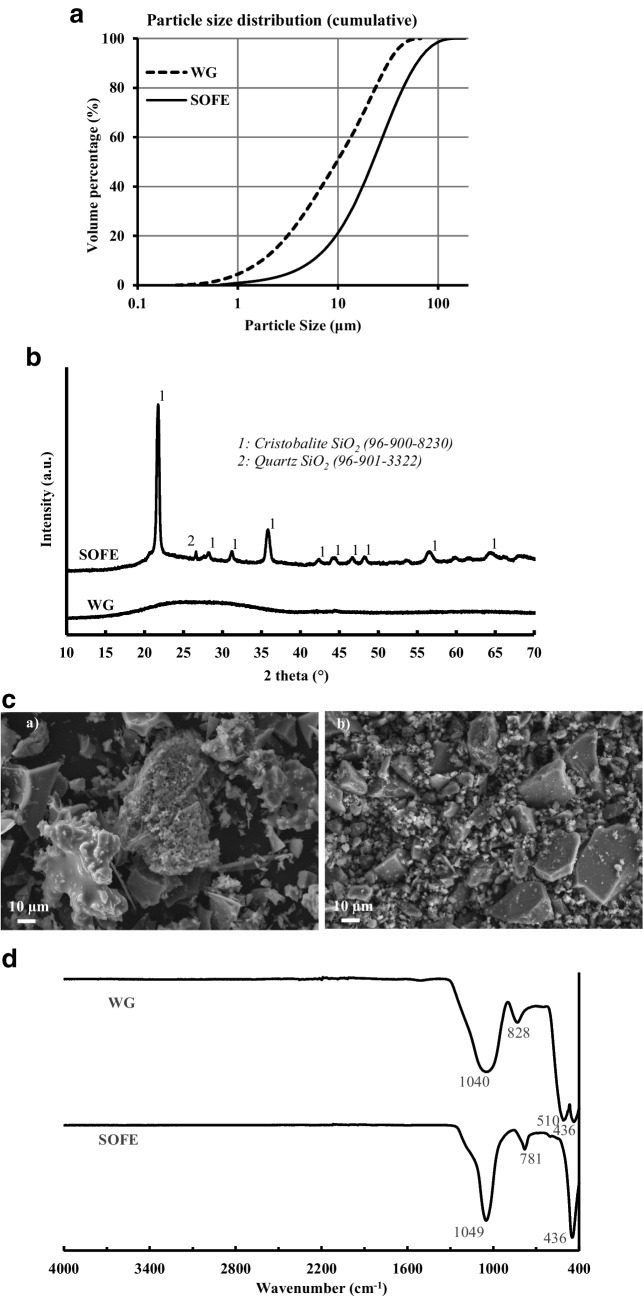
**a** Cumulative particle size distribution of raw materials, **b** X-ray diffraction patterns of SOFE and WG raw materials, **c** SEM images of SOFE a) and WG b) raw materials (× 600) and **d** FTIR spectra of SOFE and WG raw materials

The calcined material underwent a milling and homogenization process lasting for 8 h in a ball mill. Subsequently, it was sieved to attain a particle size of less than 0.100 mm. The final particle size distribution of the raw material was assessed utilizing a Mastersizer 2000 analyzer manufactured by Malvern Instruments, and the outcome of this analysis is shown in Fig. 3a. The results yielded a *D*_50_ value of 28.7 µm, with 97% of particles below the nominal sieving size of 0.100 mm. The chemical composition of the pretreated residue was analyzed by X-ray fluorescence (XRF), using an equipment Zetium Malvern Panalytical (USA). Results exhibited in Table 1 show that SOFE is mainly composed of SiO_2_ and Al_2_O_3_, with minor amounts of Na_2_O, Fe_2_O_3_ and other elements. Mineralogical phases of SOFE residue were studied by the X-ray diffraction technique with an equipment Empyrean X-ray powder diffraction with a PIXcel-3D detector of PANanalytical (Malvern, UK). The analysis was performed from 2θ = 10° to 2θ = 70° with a step size of 0.13, and the operation parameters were set at Cu K radiation, 0.15418 nm, 45 kV and 40 mA. The results, presented in Fig. 3b, showed that the main mineralogical phase in SOFE is cristobalite (96–900-8230), while quartz (96–901-3322) appears as a minor component. The microstructure of the residue was also studied by the scanning electron microscopy (SEM) technique using a JEAL SM 840 model (Akishima, Tokyo, Japan) assisted by energy dispersive X-ray spectroscopy (EDS). The sample was carbon coated utilizing a JEOL JFC 1100 sputter coating. The micrograph obtained, exposed in Fig. 3c, shows a heterogeneous size distribution of particles with different shapes, including some milled and calcined diatomite skeletons agglomerated with the corresponding microporosity associated with these structures. The study of the functional groups was carried out by using a Bruker Vertex 70 Fourier-transform infrared (FTIR) spectrophotometer (Billerica, MA, USA) spectrophotometer in the range from 400 to 4000 cm^−1^ (Fig. 3d). The SOFE residue, mainly composed of crystalline phases quartz and cristobalite, presents the expected peaks corresponding to Si–O-(Si,Al) bonds asymmetric and symmetric stretching vibration at 1049 cm^−1^ and 781 cm^−1^, respectively. Additionally, the peak at 436 cm^−1^ is attributed to the bending vibration of these bonds. The lack of any peak or band over 1300 cm^−1^ evidences the absence of water or carbonates in the sample.

**Table 1:** Chemical composition of raw materials

Material	SiO_2_	Al_2_O_3_	Na_2_O	Fe_2_O_3_	K_2_O	CaO	Ti_2_O	MgO	Other
SOFE	84.29	5.87	3.47	2.41	1.51	0.96	0.58	0.35	0.56
WG	73.93	1.00	12.12	0.42	0.71	10.59	0.04	0.51	0.68

#### Waste glass (WG)

The WG was obtained from discarded household glass bottles and containers. Foreign elements such as caps, labels and adhesive remnants were removed. Afterward, the glass elements were dried and ground in a ball mill, and the resulting powder was sieved to ensure a particle size below 0.063 mm (Fig. 3a), obtaining a powder with *D*_50_ and *D*_90_ values of 22.3 µm and 61.3 µm, respectively.

The chemical composition, particle size distribution, mineralogical phases, microstructural morphology and functional groups of waste glass were studied with the same equipment as for the SOFE residue and using the same methods. WG is mainly composed of silica and, in a lesser degree, sodium oxide and calcium oxide (Table 1). Its mineralogical structure is mostly amorphous with non-observable diffractal matter (Fig. 3b) and, microstructurally, consists of particles in a wide range of shapes and sizes as shown in Fig. 3c. The FTIR spectra (Fig. 3d) show typical peaks corresponding to Si–O-Si stretching vibrations (1040 cm^−1^ and 828 cm^−1^) and bending vibrations (510 cm^−1^ and 436 cm^−1^). The width of the peaks and their smoothed profiles underline the amorphous nature of the WG, in line with the XRD analysis results.

### Alternative solid activators synthesis

The activator was prepared through a thermochemical process as suggested in existing research (Vinai and Soutsos 2019). Various mixtures of NaOH and WG, with a small and constant quantity of water for homogenization purposes, were heated in a furnace up to 300 °C and maintained at that temperature for 3 h. The resulting solid material was subsequently pulverized into a powder using a ball mill.

The thermochemical synthesis of sodium silicate necessitates a source of sodium oxide, a source of silica, a high-pH alkaline environment and heat. In this study, the primary source of sodium oxide was sodium hydroxide pellets (Panreac, 98%), which, after stoichiometric conversion, can be assumed to consist of 75.93% wt. sodium oxide. Furthermore, the waste glass residue contributed 12.12% by weight of sodium oxide (Table 1). In the case of silicon dioxide, it is exclusively provided by the waste glass, comprising 73.93% by weight of SiO_2_ (Table 1).

To calculate the dosages of NaOH and WG, two criteria were considered. Firstly, the total weight (*M*_T_) of the solids in the initial paste to create the activator was decided to have a certain constant value in all cases. Secondly, the module of the activator, defined as the SiO_2_/Na_2_O molar ratio in the paste, was intended to have three different and established values for optimization purposes. The first assumption depends solely on the weights of NaOH and WG used, as expressed in Eq. 1. Nevertheless, the molar ratio calculation requires taking under consideration additional parameters such as the content in Na_2_O and SiO_2_ in both waste glass and commercial sodium hydroxide, their dosage and the molar weight of the species. The way these parameters are used to calculate the modulus of the activator is exposed (simplified) in Eq. 2.

1 $${M}_{{\text{T}}}= {m}_{{\text{WG}}}+{m}_{{\text{NaOH}}}$$

2 $$Ms=\frac{{\mathrm{\%}}_{{\text{WG}},{{\text{SiO}}}_{2}}\cdot {m}_{{\text{WG}}}}{{\mathrm{\%}}_{{\text{NaOH}},{{\text{Na}}}_{2}{\text{O}}}\cdot {m}_{{\text{NaOH}}}+{\mathrm{\%}}_{{\text{WG}},{{\text{Na}}}_{2}{\text{O}}}\cdot {m}_{{\text{WG}}}}\cdot \frac{{{\text{Mm}}}_{{{\text{Na}}}_{2}{\text{O}}}}{{{\text{Mm}}}_{{{\text{SiO}}}_{2}}}$$

*M*_T_: total initial mass, excluding water
*m*_x_: mass of component “*x*”
%_*x*__*,y*_: weight percentage of component “*y*” in the material “*x*”
Mm_x_: Molar mass of component “*x*”

The dosages of waste glass and NaOH required to produce the different activators were determined by solving the system formed by Eqs. 1 and 2 above. The total solid content (*M*_T_) was set at 40 g and the values of Ms were set at 0.5, 1.0 and 1.5 for the activators denoted as A0.5, A1.0 and A1.5, respectively. A consistent mass of 10 g of water was added. The mixture of sodium hydroxide previously dissolved in the water and the waste glass was manually mixed until homogenization in all cases. The calculated amounts of NaOH and WG for every mixture are presented in Table 2. During the heat treatment at 300 °C, the synthesis of sodium silicate occurred following Reaction 1. Since a portion of the hydroxyl groups, along with the added water, partially evaporated during the process, the solid obtained was weighted, and the final percentage of sodium oxide in each sample was calculated according to Eq. 3, and it is also presented in Table 2.

**Table 2:** Alternative activators initial composition and final Na_2_O percentage

Activator	WG [g]	NaOH [g]	H_2_O [g]	Na_2_O [%]
A0.5	14.039	25.961	10	56.7
A1.0	21.682	18.318	10	43.9
A1.5	26.490	13.510	10	35.9

R.1 $$2 {\text{NaOH}}+{{\text{SiO}}}_{2}\stackrel{\Delta {\text{T}}}{\to } {{\text{Na}}}_{2}{{\text{SiO}}}_{3}+ {{\text{H}}}_{2}{\text{O}}$$

3 $${\mathrm{\%}}_{{{\text{Na}}}_{2}{\text{O}}}=\frac{{\mathrm{\%}}_{{\text{NaOH}},{{\text{Na}}}_{2}{\text{O}}}\cdot {m}_{{\text{NaOH}}}+{\mathrm{\%}}_{{\text{WG}},{{\text{Na}}}_{2}{\text{O}}}\cdot {m}_{{\text{WG}}}}{{{M}{\prime}}_{{\text{T}}}}\cdot 100$$

%_x__,y_: weight percentage of component “*y*” in the material “*x*”
*m*_x_: mass of component “*x*”
*M*’_T_: total activator mas, after heat treatment

The alternative solid activators obtained, prior to being employed in the manufacture of AAMs, were ground in a ball mill and reduced into powder. A Retsch PM100 planetary mill was used for 3 min at 300 rpm for this purpose.

### Sample manufacturing and description

One-part AAMs were manufactured just by adding distilled water to a mixture of precursor and activator previously prepared. The amount of water for every sample was calculated according to 0.9 g H_2_O/g SOFE plus 0.25 g H_2_O/g activator to maintain constant workability. The resulting pastes were homogenized in a planetary mixer for 90 s, poured into moulds, and subjected to 60 strokes on a punching table to achieve the settlement of the paste. After 24 h, samples were demoulded and kept at ambient temperature. The addition of the activator was calculated, according to its Na_2_O content (Table 2), to achieve values of 10, 20 and 30 g of Na_2_O added per every 100 g of precursor. A batch of samples activated with commercial sodium silicate and sodium hydroxide mixture was also created to compare with the best of alternatively activated prototypes. The utilization of specific steel prismatic moulds allowed the manufacturing of 6 identical samples for each batch with nominal measures of 10 mm × 10 mm × 60 mm. Additionally, two plate-shaped samples were specially created in order to determine the thermal conductivity of the AAMs.

**Fig. 4:**
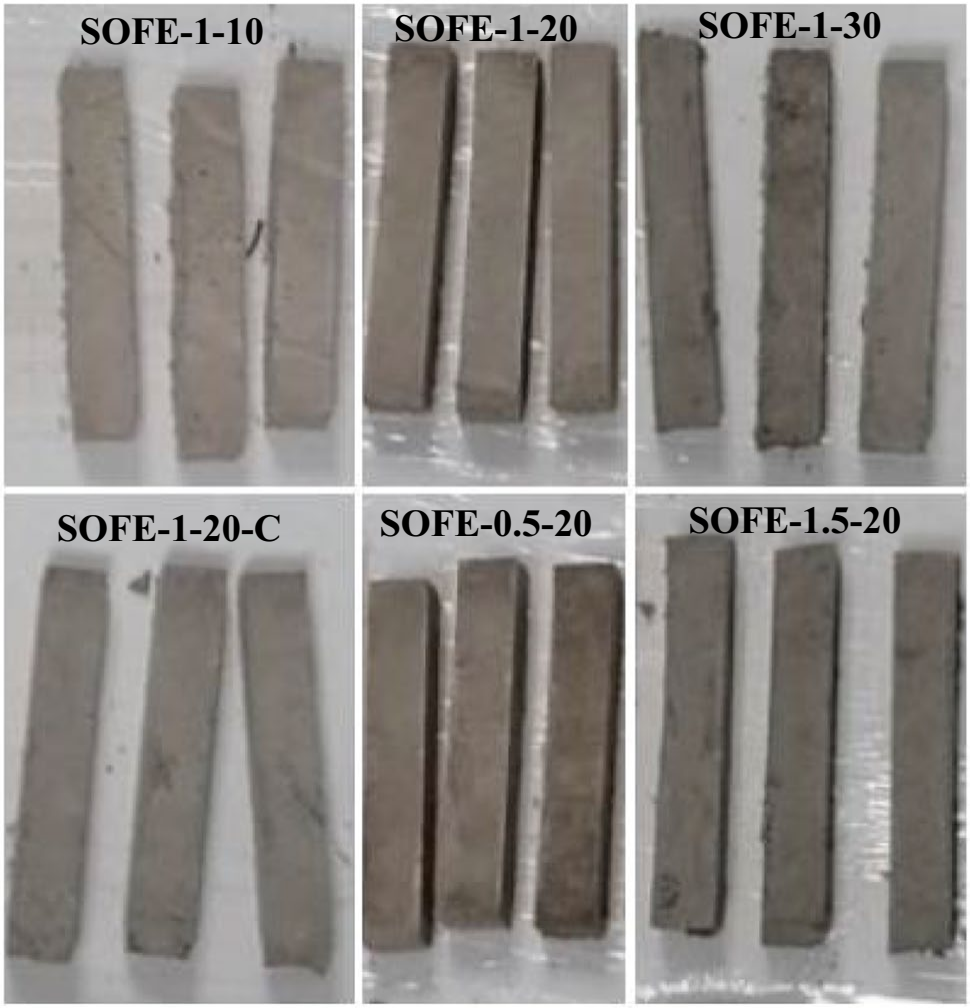
AAM specimens manufactured

As shown in Tables 3 and 4, the samples were designated as SOFE-x–y, where “x” is the module Ms of the alternative activator used and “y” corresponds to the mass of Na_2_O supplied by means of the activator per every 100 g of precursor. The control sample was named as “SOFE-1–20-C”. Figure 4 shows the specimens manufactured according to the compositions of Tables 3 and 4.

**Table 3:** One-part alternatively activated materials designation and composition

Sample data			Solid phase		Liquid phase
Name	Na_2_O [g/100 g pre]	Ms [mol/mol]	Precursor [g]	Activator [g] (name)	H_2_O [g]
SOFE-1–10	10	1	60	13.668 (A1.0)	57.417
SOFE-1–20	20	1	60	27.336 (A1.0)	60.834
SOFE-1–30	30	1	60	41.004 (A1.0)	64.251
SOFE-0.5–20	20	0.5	60	21.155 (A0.5)	59.289
SOFE-1.5–20	20	1.5	60	33.455 (A1.5)	62.364

**Table 4:** Two-part commercially activated control sample designation and composition

Sample data			Solid phase	Liquid phase		
Name	Na_2_O [g/100 g pre]	Ms [mol/mol]	Precursor [g]	NaOH (g)	SS solution (g)	H_2_O [g]
SOFE-1–20-C	20	1	60	11.131	39.840	29.339

### Sampling and test methods

Functional groups, mineralogical phases and morphological characteristics of both the manufactured activators and AAMs were investigated by FTIR spectroscopy, X-ray diffraction and scanning electron microscopy techniques, respectively. The equipment and methodologies used for these analyses were consistent with those applied to the raw materials and described in the preceding sections. The tests conducted on the samples were carried out at 28 days of curing. Until that moment, samples were kept at ambient temperature (50% relative humidity and 25 °C).

The mechanical properties were evaluated by conducting flexural and compressive strength tests following the UNE-EN 1015–11:^2020^ standard. Flexural strength for the AAMs was determined using an MTS Insight 5 machine (Eden Prairie, MN, USA) with a 5 kN capacity, employing a displacement speed of 0.2 mm/min. Compressive strength measurements were performed using a universal testing machine MST 8101 (100 kN) at a displacement rate of 2.0 mm/min.

As per physical tests, the determination of water absorption and apparent density involved an initial weighting of the specimens, followed by immersion in distilled water for a 24-h period to record both hydrostatic and saturated weights, in accordance with the Archimedes principle. The calculation of total porosity was carried out from the relationship between apparent density and true density. To determine true density, the pycnometer method was employed on finely pulverized samples with a particle size below 0.063 mm, utilizing ethanol as the displacing fluid. All of these experimental procedures were executed in compliance with the UNE-EN 1936:^2007^ standard.

For the execution of all these tests, 5 samples of each lot were initially subjected to a flexural strength test, resulting in 10 new test specimens. Subsequently, five of them underwent the compressive strength test while the remaining fragments and samples were used for physical tests and the rest of the analyses.

Additionally, the thermal conductivity tests were independently conducted on two cylindrical plate-shaped samples, at 20 ۜ°C and according to ISO 8302:^1991^ using a FOX 50 heat flow meter (TA Instruments, New Castle, DE, USA).

## Results and discussion

### Alternative solid activators characterization and optimization

#### XRD analysis of the activators

**Fig. 5:**
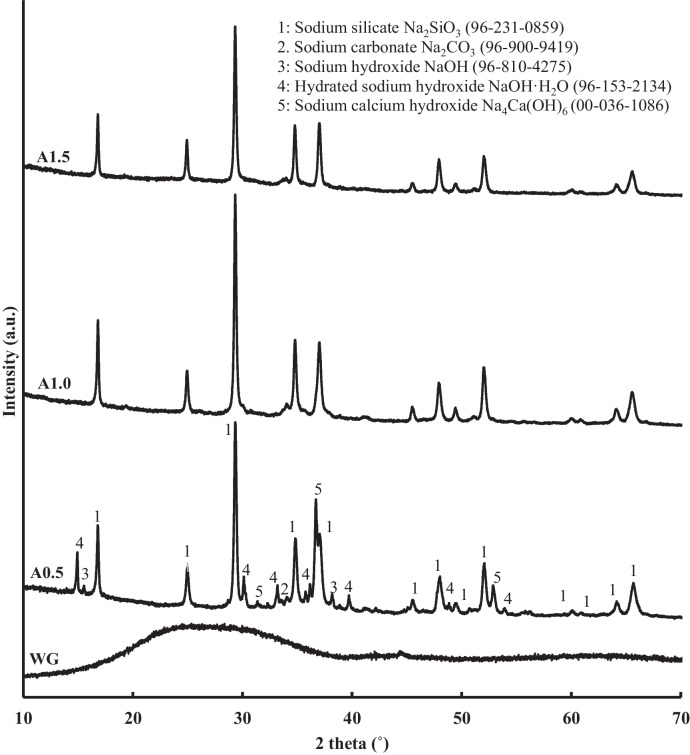
XRD patterns of alternative solid activators and waste glass raw material

According to Reaction 1, the primary and desired product derived from the heat treatment of the sodium hydroxide-waste glass mixtures should be sodium silicate. Since sodium silicate presents a mainly crystalline structure, X-ray diffraction (XRD) was one of the techniques employed to evaluate the suitability of the alternative solid activators produced. Figure 5 exposes the XRD patterns of waste glass raw material and the alternative activators produced.

In contrast with the amorphous raw material, all three activators exhibit the characteristic peaks of sodium silicate at 2θ = 17°, 29.6°, 35.1°, 37.4° and others, along with a minor presence of sodium carbonate with a peak at 2θ = 33.9°. Sodium silicate–related peaks are especially well-defined and with certainly high intensity in activator A1.0. Furthermore, XRD patterns present two noteworthy trends that deserve attention. On one hand, the activator A1.5 diffraction pattern exhibits less intense peaks, suggesting a lower formation of crystalline sodium silicate. On the other hand, the major content of NaOH in activator A0.5 did not lead to a greater formation of sodium silicate but to the appearance of crystalline sodium-calcium hydroxide as a secondary reaction product and the presence as well of unreacted anhydrous sodium hydroxide NaOH at 2θ = 15.6° and 38.2°, and also the monohydrated form NaOH·H_2_O at 2θ = 14.8°, 35.8° and 29.9°.

Kikuchi and Koga (2022) reported that a mixture of CaO, NaOH and Ca(OH)_2_ is stable under certain conditions below 600 K. In this sense, the hybrid sodium-calcium hydroxide is considered to be formed as a result of the excess of NaOH incorporated into this sample in relation to the amount stoichiometrically necessary according to Reaction 1. In the first stage, the elevated pH of the paste provokes the dissolution of part of the amorphous CaO from waste glass. Subsequently, the results of XRD analysis (Fig. 5) suggest that during the heat treatment at 300 °C, Reaction 2 takes place and the sodium and calcium hydroxides crystalize together to form the hybrid hydroxide as can be deduced from the peaks at 2θ = 31.5°, 36.6° and 52.7°. The absence of these peaks in samples A1.0 and A1.5 can be attributed to the lesser amount of NaOH present in the mixture in order to obtain the desired modulus of 1.0 and 1.5, respectively.

R.2 $$6{\text{NaOH}}+{\text{CaO}}\to 4{\text{NaOH}}+{{\text{Ca}}({\text{OH}})}_{2}+ {{\text{Na}}}_{2}{\text{O}}$$

In conjunction, the results of the XRD analysis suggest that sodium silicate synthesis is optimum when the modulus of the activator is equal to 1. It can be seen that sample A1.0 showed a major presence of sodium silicate with a minor appearance of secondary reaction products. Additionally, sample A1.5 with slightly more attenuated peaks still seems competitive with a total absence of secondary products as well. On the contrary, according to XRD analysis results, the A0.5 sample should be rejected as the objective of this stage of the research is maximizing the quantity of sodium silicate synthetized.

In other studies, mixtures of NaOH and the residue with SiO_2_ are often made in proportions that do not adhere to chemical or stoichiometric criteria (Dadsetan et al. 2022; Marin et al. 2023). In this case, manufacturing activators with specific modules using the created equations allows for selecting the optimal module rather than adhering strictly to proportions, which may vary between materials. In contrast, the approach of selecting optimal modules for activators could be extended to the utilization of other residues.

#### FTIR analysis of the activators

**Fig. 6:**
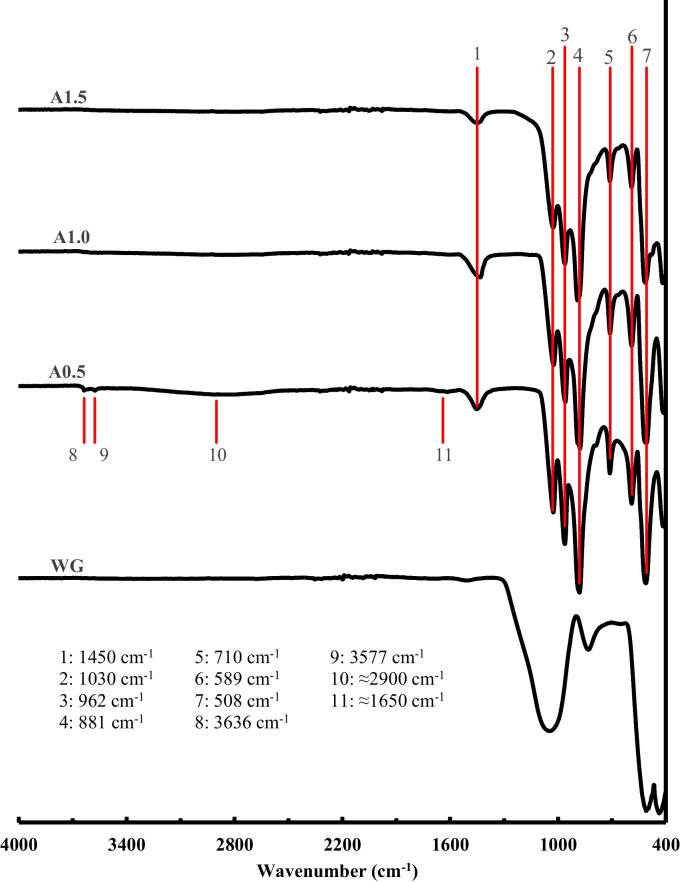
FTIR spectra of synthetized activators, as a function of Ms module

FTIR analysis was carried out in order to study the new structures formed in the activators in comparison with WG raw material. The results of this study are exposed in Fig. 6.

As previously explained, during the treatment at elevated pH and temperature, Reaction 1 takes place and sodium silicate is synthetized. It leads to the disruption of the [-Si–O-Si-] network found in the waste glass, with sodium being integrated as [-Si–O-Na] (Ferraro and Manghnani 1972; Serra et al. 2003). Consequently, this reaction results in the formation of non-bridging oxygens bonded to silicon atoms. Because the reaction mechanism and the manner in which sodium is integrated occur randomly, this phenomenon results in the presence of silicon atoms with varying degrees of connection to non-bridging oxygens. This, in turn, causes the main typical peak associated with Si–O bonds to split into three distinct peaks, observable at 1030 cm^−1^, 962 cm^−1^ and 881 cm^−1^. These peaks are characterized by their narrow and well-defined nature, consistent with the findings of the XRD analysis, which indicated that the synthesized sodium silicate exhibits a crystalline structure. The presence of non-bridging oxygens enhances a better dissolution of silicon species improving its availability to form other compounds, which is an extremely interesting characteristic (Jansson et al. 2015; Pignatelli et al. 2016). In fact, it could explain the high reactivity of the activators produced, since they are able to supply silicon that can be rapidly dissolved, being available for activation reactions (Vinai and Soutsos 2019). The peak at 1450 cm − ^1^ is attributable to the C–O bond owing to the carbonation phenomena that, as expectable, affects more pronouncedly to the A.05 sample due to the presence of unreacted NaOH, which tends to carbonate in contact with air. In this sense, the broad band identified at 2900 cm^−1^ for the A.05 sample but barely perceivable for the A1.0 sample and almost inexistent in the A1.5 activator is also considered indicative of the presence of sodium carbonate (Vinai and Soutsos 2019). Furthermore, the A0.5 sample showed peaks at 3636 cm^−1^ and 3577 cm^−1^ which highlights the presence of O–H bonds from hydroxide groups (Navaneetha et al. 2023). Specifically, the peak at 3577 cm^−1^ is considered to be related to O–H bounds from NaOH while the peak at 3636 cm^−1^ can be attributed to O–H bounds from Ca(OH)_2_ structures (Wang et al. 2020) in correlation with the presence of the hybrid sodium-calcium hydroxide observed in the results of XRD analysis for this sample.

#### SEM analysis

**Fig. 7:**
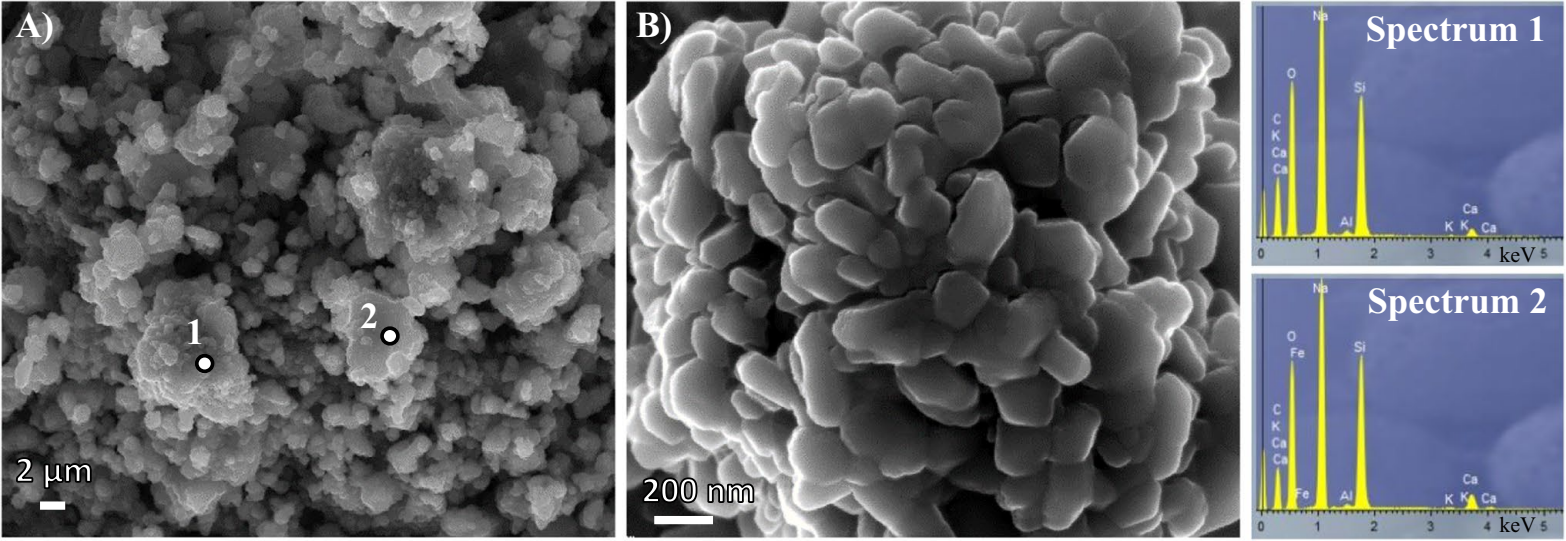
SEM images of A1.0 activator at 2 kx (**A**) and 45 kx magnification (**B**). EDX analysis (spectra 1 and 2)

Figure 7 shows the microstructure of the optimum manufactured alternative solid activator A1.0. In contrast with WG raw material (Fig. 3c), these images show quite a homogenous structure, where small crystalline structures can be noticed especially well at 45 kx magnification. This homogeneity and crystallinity correlate with the results of the XRD analysis of the activators.

EDX analysis, spectrum 1 and spectrum 2, revealed quite a homogeneous composition with an abundant presence of sodium, silicon and oxygen, which aligns with the production of sodium silicate according to Reaction 1 and previously observed in FTIR and XRD analysis results.

### AAMs characterization

#### Mechanical properties

**Fig. 8:**
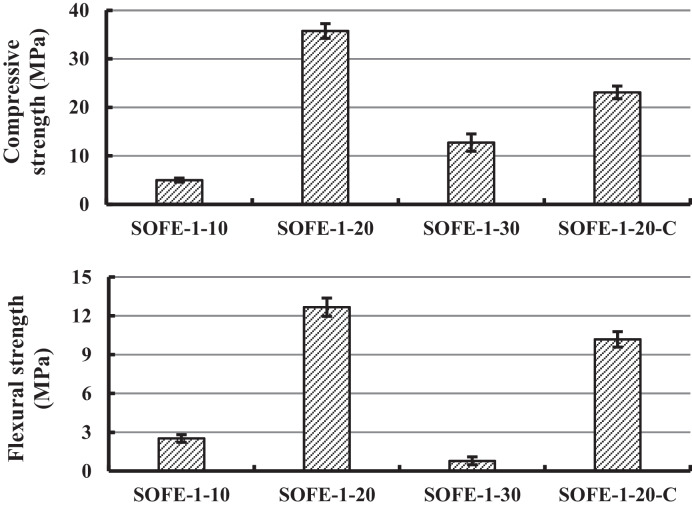
Influence of activator dosage on compressive and flexural strength at 28 days of curing

Figure 8 displays the results obtained from the compressive and flexural strength tests of one-part samples as a function of the quantity of added Na_2_O through the alternative activator with a modulus of 1 (A1.0). It also includes the two-part control sample, activated using a mixture of commercial NaOH and sodium silicate with an equivalent modulus and the same Na_2_O amount as the optimal alternative activator addition.

The results of both compressive and flexural strength tests provide a comprehensive overview of the mechanical behaviour of the specimens manufactured after 28 days of curing. Reference values offered by the results of the SOFE-1–20-C sample made with commercial activators were 23.1 MPa and 10.2 MPa for compressive and flexural strength, respectively. Sample SOFE-1–20 exhibited excellent mechanical characteristics, displaying a remarkable compressive strength of 35.8 MPa and a notable flexural strength of 12.7 MPa, outperforming the control sample significantly in both parameters. In contrast, sample SOFE-1–10 showed lower strengths, with a compressive strength of 5.0 MPa and a flexural strength of 2.5 MPa owing to an inefficient activation process provoked by the insufficient supply of activator, which led to a deficient structure, with unreacted precursor particles as registered during microstructural analysis. Sample SOFE-1–30 presented intermediate mechanical behaviour, with a still competitive compressive strength of 12.7 MPa but with an unusually low flexural strength of 0.8 MPa. These unbalanced strength results in sample SOFE-1–30 are explained by the existence of a compact structure with a certain amount of gel but with the presence of cracks distributed all along the sample as discussed in the subsequent section of SEM analysis where relating images are exposed (Figs. 15 and 16). Therefore, a higher alkali dosage (30 g Na_2_O /100 g SOFE) is unfavourable for the mechanical properties of the SOFE resulting in less amorphous gel possibly due to the rapid formation of oligomers covering the surface, which will prevent the alternative activator from further dissolving inside the SOFE, decreasing the degree of reaction.

**Fig. 9:**
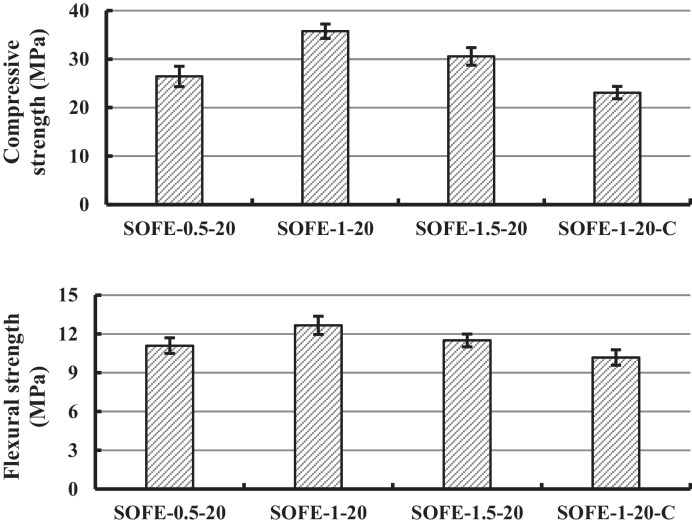
Influence of activator modulus on compressive and flexural strength at 28 days of curing

The analysis of the results displayed in Fig. 8 also exposes the elevated influence of the quantity of alternative activators added on the mechanical properties of the specimens, even surpassing those of the reference commercially activated sample when the dosage is optimal. On the contrary, the variation in the activator modulus while keeping the addition of Na_2_O constant (using activators A0.5 and A1.5) was found to have a relatively minor influence on the mechanical properties of the samples, as can be observed in Fig. 9.

Sample SOFE-0.5–20 remained quite competitive to the mechanically superior sample SOFE-1–20 even when the activator A0.5 showed to have a lower content in sodium silicate. Nevertheless, the excess of unreacted NaOH in this activator, already discussed in XRD and FTIR sections, could facilitate a major dissolution of silicon from the precursor, compensating the previous inconvenience and leading to mechanical strength not so far from the maximum achieved. In sample SOFE-1.5–20, the superior modulus of the activator A1.5 and the inferior content in sodium silicate unexpectedly led to just slightly lower compressive and flexural strength in comparison with optimal sample SOFE-1–20. This behaviour is attributed to the contribution of nanosilica in excess from the activator to mechanical strength. It has been suggested that nanosilica may contribute to compacting the structure owing to its capacity to fill spaces, and additionally, it holds a high pozzolanic activity (Matos and Sousa-Coutinho 2012; Lu et al. 2020).

It is noteworthy to highlight that, when the addition of Na_2_O is maintained constant at 20 g of Na_2_O per every 100 g of precursor, all alternative activated samples surpassed the mechanical strength of commercially activated control sample SOFE-1–20-C.

#### Physical properties

The influence of the amount of Na_2_O supplied by means of the optimum alternative activator dosage on the physical properties of the manufactured specimens is exposed in Table 5 and compared with the control sample (SOFE-1–20-C). Furthermore, Table 6 shows the same properties for AAMs produced with the remaining activators and a comparison with the optimum sample (SOFE-1–20) and control sample.

**Table 5:** Physical properties of AAMs produced with different Na_2_O dosages of optimum activator and control sample

SAMPLE	Bulk density [kg·m^−3^]	Water absorption [%]	Total porosity [%]
SOFE-1–10	952 ± 6	44.3 ± 1.1	54.5 ± 0.3
SOFE-1–20	1213 ± 7	21.2 ± 0.8	43.1 ± 0.2
SOFE-1–30	969 ± 28	31.7 ± 0.9	49.4 ± 0.1
SOFE-1–20-C	1118 ± 15	27.3 ± 1.0	47.2 ± 0.1

**Table 6:** Physical properties of AAMs produced with different alternative activators at constant Na_2_O dosage and control sample

SAMPLE	Bulk density [kg·m^−3^]	Water absorption [%]	Total porosity [%]
SOFE-0.5–20	1124 ± 9	25.1 ± 1.0	46.2 ± 0.2
SOFE-1–20	1213 ± 7	21.2 ± 0.8	43.1 ± 0.2
SOFE-1.5–20	1187 ± 13	24.5 ± 0.7	45.6 ± 0.2
SOFE-1–20-C	1118 ± 15	27.3 ± 1.0	47.2 ± 0.1

The physical properties determined for both commercial and alternatively activated specimens revealed correlating results, with the higher apparent density samples presenting lesser water absorption and lower total porosity as could be expected. In correlation with mechanical test results, samples with the same Na_2_O dosage (Table 6) present more similarities than those manufactured maintaining constant the activator and its modulus (Table 5). Bulk density varies across the range of 952 up to 1213 kg/m^3^ for samples SOFE-1–10 and SOFE-1–20, respectively. These results can be considered certainly interesting owing to their low values, similar to those of lightweight materials (Li et al. 2023; Kanagaraj et al. 2023). Considering these physical properties in conjunction with mechanical test results discussed in the previous section, the optimum sample significantly outperforms the control sample, with a compressive strength 55% greater, while the bulk density is only 8% higher. Water absorption ranges between 21.2 and 44.3% in correlation with total porosities of 43.1% and 54.5%, respectively. The relatively high total porosity is considered to be owing to the elevated amount of water necessary to obtain an adequate workability of the pastes (Tables 3 and 4). The total porosity is also in accordance with the results of XRD and FTIR analysis that will be discussed in subsequent sections. Those studies showed a high degree of carbonation of the samples which could be provoked by these elevated values of total porosity and, in this way, permeability to ambient air to produce the carbonation phenomena.

#### FTIR analysis

**Fig. 10:**
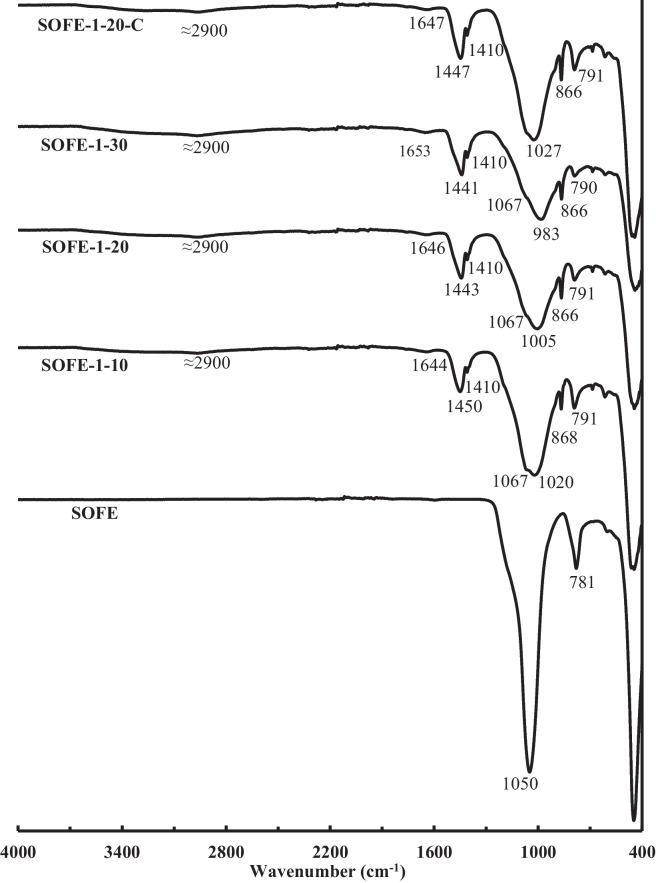
FTIR spectra of AAMs produced with different Na_2_O dosages of optimum activator and comparison with raw material and control sample

Figure 10 shows the FTIR spectra of the samples made with the optimal alternative solid activator, as a function of the dosage and a comparison with the control sample SOFE-1–20-C.

SOFE raw material is mainly composed of the cristobalite phase (Fig. 3b). The main peak associated with Si–O-Si asymmetric stretching vibrations in high-purity cristobalite is usually located at 1106 cm^−1^ (Tang et al. 2015) but in this case, it appears at a lower wavenumber of 1050 cm^−1^, which can be attributed to the presence of alumina in the raw material (Kani and Allahverdi 2009). The same phenomenon is responsible for the progressive shifting into lower wavenumbers of the new peak appearing associated with the asymmetric stretching vibration of Si–O-(Si, Al) tetrahedral bonds in geopolymer samples. This peak can be observed at decreasingly lower frequencies, from 1020 to 983 cm^−1^, as aluminium contained in cristobalite migrates into the gel structure owing to the partial dissolution of this phase as more activator is added. A similar tendency can be observed for the peak appearing between 781 and 790 cm^−1^, attributed to the symmetric stretching vibration of Si–O-Si bonds (Nabil et al. 2018; Szabó et al. 2023).

In contrast, the peak representing Si–O-Si bonds in raw material (1050 cm^−1^) shifts into higher wavenumbers owing to the loss of aluminium (1067 cm^−1^). It can be noticed how this peak is dissolved as more activator is added, so it is barely perceivable for sample SOFE-1–10 and becomes just a smooth shoulder for increased additions.

All specimens exhibit similar degrees of carbonation. Peaks relating to stretching vibrations of C–O bonds from carbonate ions bonded to sodium appear at around 1450 cm^−1^. Peaks at 1410 cm^−1^ correspond to carbonates not interacting with metal ions or just slightly influenced by them (Fine and Stolper 1986). Besides, a C–O bond bending vibration peak appears at 866 cm^−1^ (Hassan et al. 2019; Lecomte et al. 2006; Garcia-Lodeiro et al. 2009).

The smooth broad band that appears centred around 2900 cm^−1^ is also considered related to the presence of carbonation species (Vinai and Soutsos 2019). It should be noted that this C–O-related band appears partially overlapped with the band related to stretching vibration of O–H bonds in water molecules that can be observed approximately between 3200 and 3600 cm^−1^ with overtones at 1644–1653 cm^−1^ (Puertas et al. 2011; Jurado-Contreras et al. 2022) owing to the presence of adsorbed water, typical in these materials.

Control sample SOFE-1–20-C presents similar peaks and bands to alternatively activated samples. The FTIR spectrum of this sample, considering the shape, intensity and displacement of the peaks, suggests that the microstructure of SOFE-1–20-C could be at some point between samples SOFE-1–10 and SOFE-1–20 but closer to the second one. This suggestion certainly correlates with the results of mechanical strength discussed in the section above and exposed in Fig. 8.

**Fig. 11:**
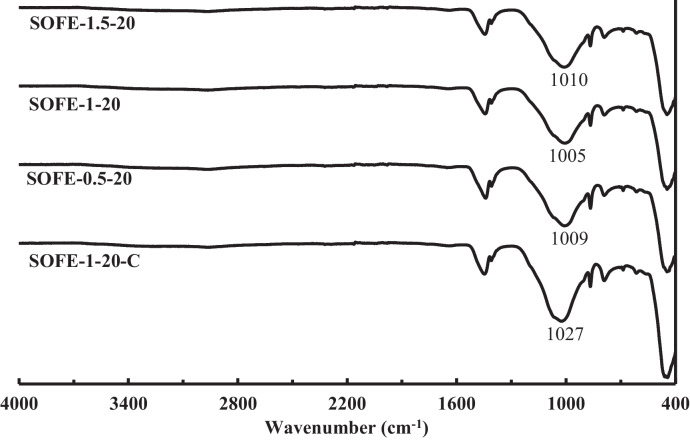
FTIR spectra of AAMs produced using alternative activators with different modulus at constant Na_2_O dosage and comparison with control sample

Figure 11 exhibits the spectra of the samples SOFE-0.5–20 and SOFE-1.5–20, manufactured with the non-optimum alternative activators (A0.5 and A1.5, respectively) compared with the optimum sample SOFE-1–20 and the control sample SOFE-1–20-C, maintaining a constant dosage of Na_2_O by means of activator. In this case, most peaks and bands are quite similar to those described in previous Fig. 10. However, the study of the displacement of the main peak still reveals additional valuable information. The first noteworthy aspect to take into consideration is that the shifting of the main peak between different samples in this case is moderately lower, which suggests a relatively higher similarity among them, in correlation with the mechanical test results discussed in the previous section.

The displacement of this peak is considered a qualitative indicator of the degree of activation. In this sense, the SOFE-1–20 sample should present the best microstructure, followed by both SOFE-1.5–20 and SOFE-0.5–20 samples. Furthermore, all samples should be superior to the control sample SOFE-1–20-C. It exactly matches with the results of mechanical strength tests exposed in Fig. 9.

#### XRD analysis

**Fig. 12:**
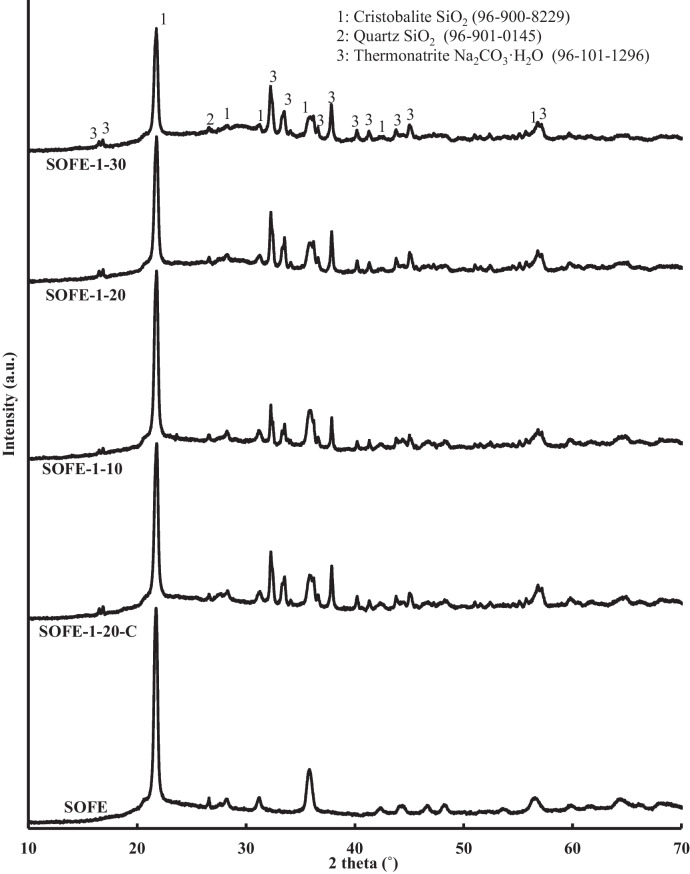
XRD patterns of AAMs produced with different Na_2_O dosages of optimum activator and comparison with raw material and control sample

Figure 12 exhibits the X-ray diffraction (XRD) patterns of specimens manufactured using the optimal alternative solid activator in different dosages and the control sample SOFE-1–20-C.

The XRD analysis indicated that the main peaks associated with the cristobalite phase (2θ = 21.8°, 2θ = 36.0°) in the raw material are still evident in the AAMs produced, although with varying levels of intensity. A tendency easily perceivable in the alternatively activated specimens, samples SOFE-1–10 to SOFE-1–30, is the attenuation of these peaks as the dosage of the activator increases. It is provoked by the gradual dissolution of this phase as more activator is used, in correlation with the results of FTIR characterization. According to XRD analysis results, the carbonation processes observed in the results of the FTIR analysis are now certainly evidenced by the presence of the phase thermonatrite (96–101-1296), which is a monohydrated crystalline phase of sodium carbonate with characteristic peaks at 2θ = 32.8°, 37.8°, 56.7° and others. In relation to the control sample SOFE-1–20-C, the intensity of the peaks related to cristobalite for this sample falls in the range between SOFE-1–10 and SOFE-1–20, in accordance with the results of FTIR analysis which correlates as well with the mechanical performance of this control sample, as explained in previous sections.

**Fig. 13:**
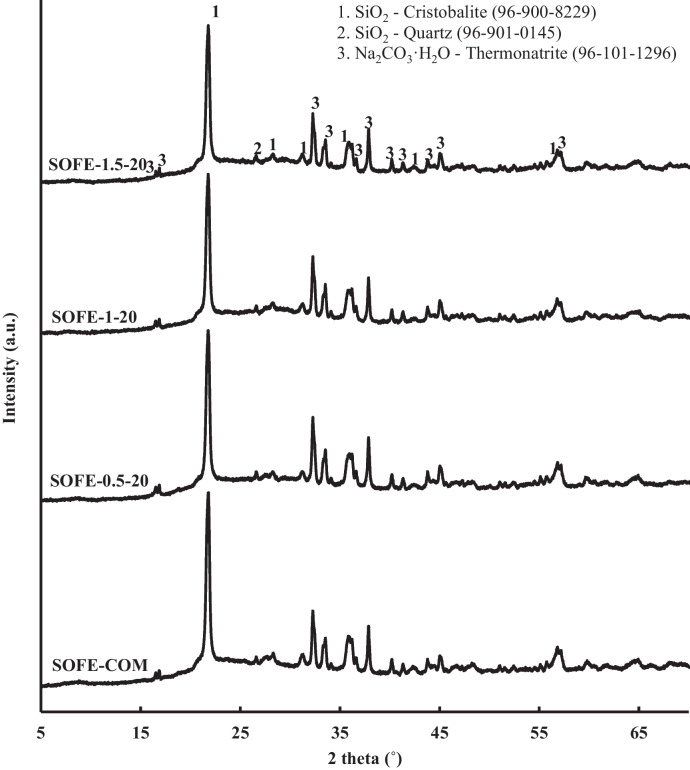
XRD patterns of AAMs produced using alternative activators with different modulus at constant Na_2_O dosage and comparison with control sample

In contrast, when the activator modulus varies while maintaining a constant Na_2_O addition, the distinctions among alternative activated samples appear to diminish, and the phases exhibit a high degree of similarity, as illustrated by the diffraction patterns in Fig. 13. Still, the control sample is discernible for clearly presenting a minor degree of cristobalite dissolution. In this sense, the reactiveness of the alternative solid activators, independently of their modulus, seems to have an important role in the dissolution of silicates present in the precursor.

#### Thermal conductivity

**Fig. 14:**
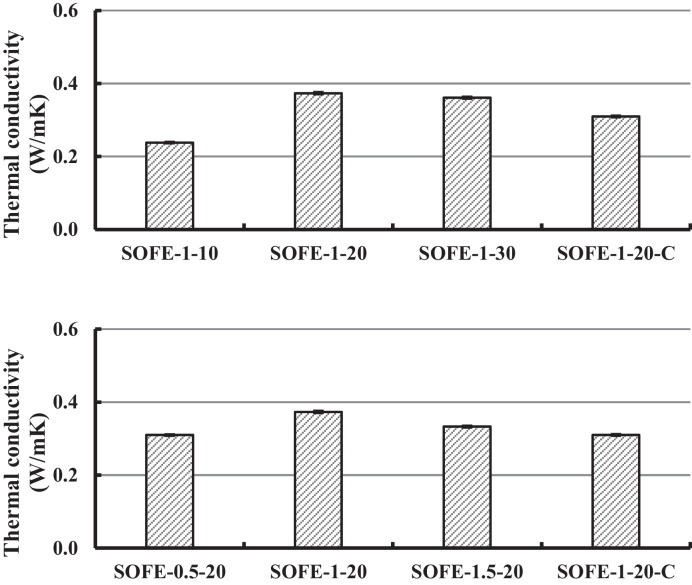
Thermal conductivity at 28 days of curing of AAMs produced with different Na_2_O dosages of optimum activator (up) and with different alternative activators at constant Na_2_O dosage (down) and comparison with the control sample

Figure 14 exhibits the results of the thermal conductivity test carried out on all manufactured samples.

The first noteworthy general aspect is the relatively low thermal conductivity for all samples, quite inferior to other ordinary binders such as Portland cement (0.8–1.2 W/mK). The values of thermal conductivity show a strong correlation with the results of physical results (Tables 5 and 6) since denser and more compact samples also present slightly higher values of thermal conductivity. Nevertheless, the differences among samples are certainly reduced, ranging from 0.24 W/mK for SOFE-1–10 to 0.37 W/mK for optimum SOFE-1–20 sample. The control sample SOFE-1–20-C showed a moderately lower thermal conductivity of 0.310 W/mK, in accordance with the results of physical properties as well.

#### SEM analysis

**Fig. 15:**
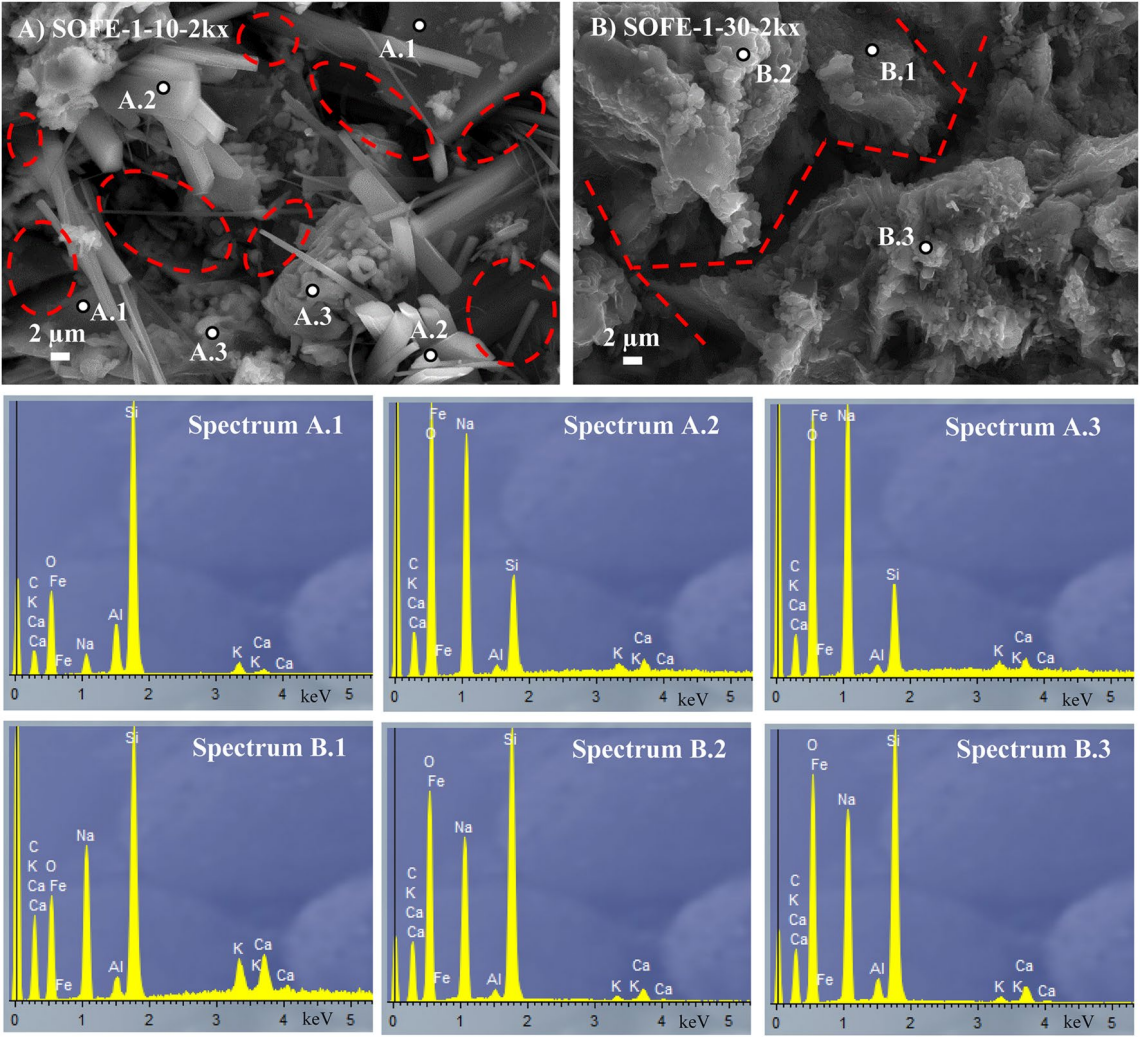
SEM images of samples SOFE-1–10 (**A**) and SOFE-1–30 (**B**) and EDX analysis (Spectra A.1 to A.3 and B.1 to B.3, respectively)

One-part samples manufactured with different dosages of Na_2_O, using the optimal alternative activator (modulus Ms = 1.0), were studied at 28 days of curing and compared with reference two-part sample commercially activated SOFE-1–20-C. Figure 15 displays the images of the materials with 10 and 30 g Na_2_O per every 100 g of precursor SOFE, as examples of defect and excess activator. Both samples showed reduced mechanical properties, as previously exposed (Fig. 8), in comparison with the optimum sample SOFE-1–20.

In correlation with the rest of the characterizations, the SOFE-1–10 sample showed relatively abundant empty cavities, responsible for its low density and high porosity in conjunction with its poor mechanical performance. Furthermore, EDX results and visual exam revealed the rest of the activator (Spectrum A.2) and unreacted particles (Spectrum A.1) owing to an incomplete process of activation. A certain amount of gel was also modestly observed at some points (Spectrum A.3). Sample SOFE-1–30 SEM analysis also aligns with the results of mechanical and physical tests. This sample presents some parts really compact and considered responsible for its high compressive strength but also open and intercommunicate cavities forming relatively wide cracks, which leads to reduced flexural strength. Sample SOFE-1–30 was made with the highest amount of alternative activator, so, in this case, calcium from waste glass (Table 1) presents as a residual element in the activator that contributed to the created geopolymeric gel with varying inclusion of calcium. Spectrum B.1 shows a gel with a relatively high content of calcium while Spectrum B.2 exposes a more globular Na-based geopolymeric gel. Spectrum B.3 would be an intermediate state according to the EDX analysis results.

**Fig. 16:**
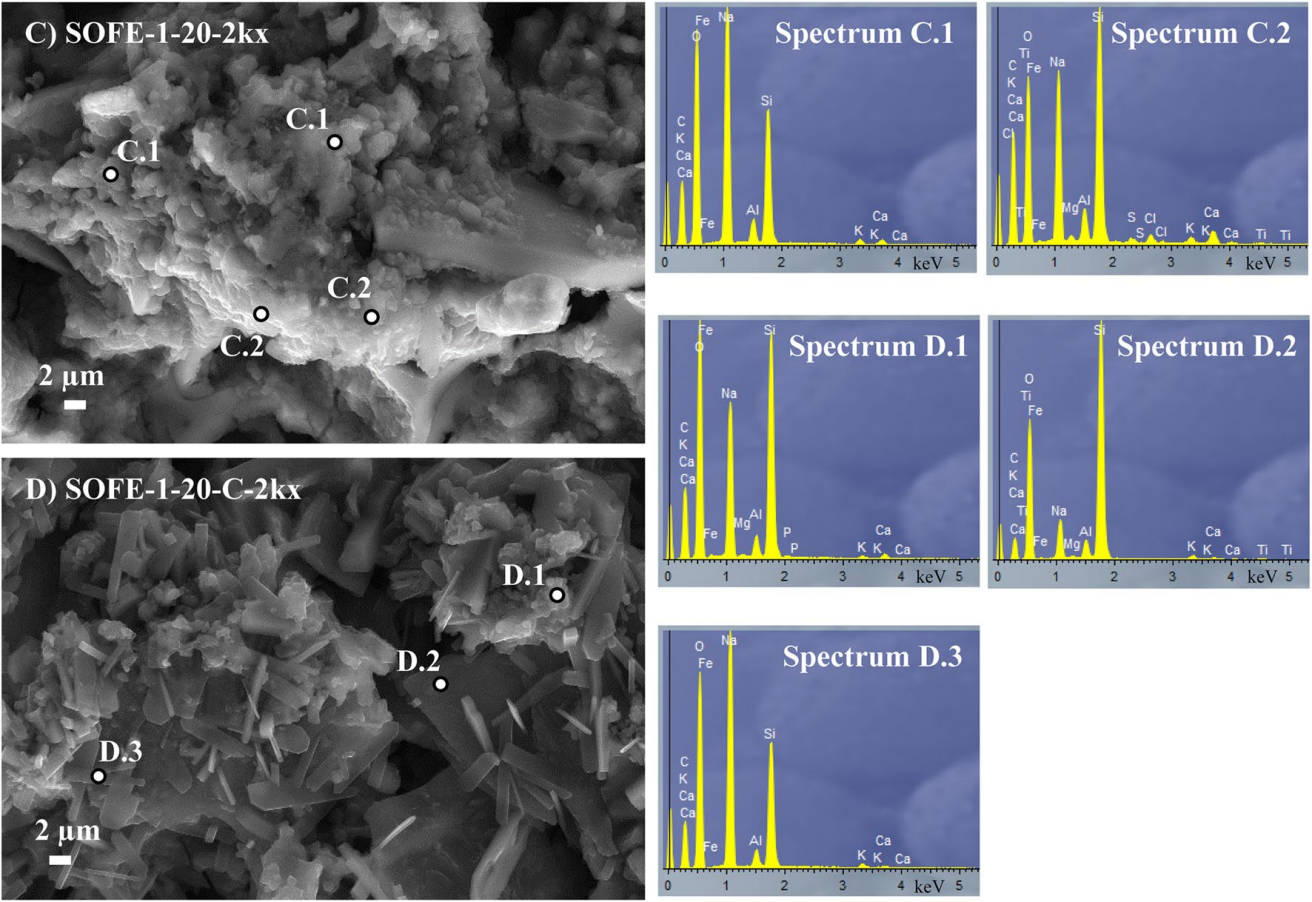
SEM images of optimal sample SOFE-1–20 (**C**), reference sample SOFE-1–20-C (**D**) and EDX analysis (Spectra C.1 and C.2 and D.1 to D.3, respectively)

Figure 16 exposes the SEM images and EDX analysis of the optimum one-part alternatively activated sample SOFE-1–20 and the two-part reference sample SOFE-1–20-C.

Sample SOFE-1–20 exhibits quite a compact structure with abundant gel-filling cavities and covering the surface of unreacted precursor particles which matches with the reported physical and mechanical properties. The aspect of the structure is certainly compact in most parts, which led to improved mechanical properties and also a slightly higher density and thermal conductivity, as explained in Fig. 8, Table 5 and Fig. 14, respectively. The composition and morphology of the abundant appearing geopolymeric gel varies from a mainly sodium-based globular gel represented by Spectrum C.1 to a less globular gel with a certain inclusion of calcium described by Spectrum C.2 As suggested by FTIR and XRD analysis previously discussed, sample SOFE-1–20-C presents a microstructure and morphology between SOFE-1–10 and SOFE-1–20 samples, in correlation as well with the mechanical performance of this sample. In this sense, in contrast with the presence of gel (Spectrum D.1) unreacted particles according to Spectrum D.2 can be observed, as well as activator crystallized structures represented by Spectrum D.3.

## Conclusions

This research represents a significant advancement in designing and dosing alternative solid activators for creating alkali-activated materials via one part. The present research introduces calculation methods and procedures for designing activators with varying modulus based on chemical and stoichiometric criteria. This also allows for precise dosing of the activators to ensure a specific contribution of Na_2_O to the activation process.

1. Fourier transform infrared spectroscopy (FTIR) has proven useful in identifying unreacted excess sodium in the thermochemical synthesis of solid alternative activators through the appearance of bands associated with O–H bonds. Additionally, X-ray diffraction is suitable for identifying the produced sodium silicate and confirming the absence of other crystalline byproducts. The combined use of both techniques facilitated the selection of the optimal activator composition (Ms = 1).
2. SOFE residue was utilized to produce AAMs with enhanced mechanical properties. The best mechanical properties were shown by the sample manufactured with the optimal activator (Ms = 1) and a dosage of 20 g of Na_2_O per 100 g of precursor. This sample achieved compression and flexural strengths of 35.8 MPa and 12.7 MPa, respectively, surpassing those of the control sample produced with commercial activators, which achieved 23.1 MPa and 10.2 MPa, respectively.
3. All geopolymers manufactured showed low conductivity values, with a maximum of 0.37 W/mK for sample SOFE-1–20, widely inferior to Portland cement (0.8–1.2 W/mK).
4. XRD and FTIR analysis of the AAMs revealed structures with different degrees of activation suggesting microstructural differences. The microstructure of the samples is widely influenced by the activation conditions and correlates with the mechanical performance of the samples. In particular, geopolymeric gel with varying composition and degrees of calcium integration was found.

Globally, this research contributes to the use of residues to produce not only the precursor but also the activator for the synthesis of AAMs. Therefore, it opens the door to a bigger utilization of wastes in line with the current strategies of circular economy with subsequent environmental benefits.

## Data Availability

All data supporting the findings of this study are available upon reasonable request.
